# Superficial Siderosis: A Case Report of Underdiagnosed Disorder

**DOI:** 10.7759/cureus.69768

**Published:** 2024-09-20

**Authors:** Tural Talibov, Meltem Inci, Mehmet Barburoglu, Altay Sencer, Oguzhan Coban

**Affiliations:** 1 Department of Neurology, Istanbul Health and Technology University, Istanbul, TUR; 2 Department of Neurology, Istanbul Avcilar Murat Koluk Community Hospital, Istanbul, TUR; 3 Department of Radiology, Istanbul University, Istanbul Faculty of Medicine, Istanbul, TUR; 4 Department of Neurosurgery, Istanbul University, Istanbul Faculty of Medicine, Istanbul, TUR; 5 Department of Neurology, Istanbul University, Istanbul Faculty of Medicine, Istanbul, TUR

**Keywords:** cervical dural defect, progressive ataxia, progressive hearing loss, spinal csf leak, superficial siderosis

## Abstract

Superficial siderosis (SS) is caused by subpial hemosiderin deposition due to chronic low-grade bleeding into the subarachnoid space. Dural tears are the most common etiology. Slowly progressive gait ataxia and hearing impairment are common clinical manifestations. Brain magnetic resonance imaging (MRI) shows linear superficial hypointensity on the T2 weighted images and gradient echo. The therapeutic approach is surgical repair of the bleeding source.

The patient presented with progressive hearing loss and ataxia. Neurological examination revealed bilateral hearing loss, nystagmus, dysarthria, brisk deep tendon reflexes, and severe ataxia. Brain MRI showed linear superficial siderosis in the cerebrum, cerebellum, and brain stem. Spinal MRI showed ventral epidural cerebrospinal fluid (CSF) collection and disc-osteophyte complex. Six months after the surgical repair of the dural defect, the patient's neurological examination demonstrated improvement in ataxia and dysarthria. The patient was able to walk without any assistance.

Surgical repair of the underlying bleeding source may be beneficial in preventing the progression and improving the symptoms of superficial siderosis SS. This case suggests that SS symptoms are potentially reversible by surgical treatment of the underlying spinal CSF leak after a long disease course.

## Introduction

Superficial siderosis (SS) is a rare disease due to the deposition of hemosiderin in the subpial space as a result of chronic and leaky hemorrhage into the subarachnoid space. Inflammation caused by toxic free iron accumulation in the pia matter is thought to play a role in the pathogenesis [1]. Due to the limited number of cases, its prevalence remains unknown. The most common etiology of classic SS is spinal dural defects [2-4]. The most typical clinical manifestation is a slowly progressing cerebellar ataxia that is frequently accompanied by hearing loss and corticospinal tract signs [1,5]. In SS cases with a spinal dural defect, a blood patch or surgical repair of the defect has been shown to stop clinical progression and provide recovery [6,7]. Here, we report a case of SS presenting with progressive cerebellar dysfunction and hearing loss who benefited from surgical repair of the cervical dural defect.

## Case presentation

A 54-year-old male patient was admitted to our neurology outpatient clinic with a gradual onset of bilateral sensorineural hearing loss that began eight years ago and progressively worsened over time. In the past three years, he developed additional symptoms, including gait instability and dysarthria characterized by slurred speech. His medical history was unremarkable. The neurological examination revealed cerebellar dysarthria, nystagmus, and bilateral sensorineural hearing loss. Examination of the remaining cranial nerves was normal. His tendon reflexes were hyperactive in all of the limbs. The finger-nose and heel-shin tests were abnormal, with bilateral dysmetria. Additionally, he exhibited severe truncal ataxia. Superficial sensation and motor strength were normal. His gait was wide-based and unsteady, requiring assistance for ambulation. Romberg’s sign was positive.

**Figure 1:**
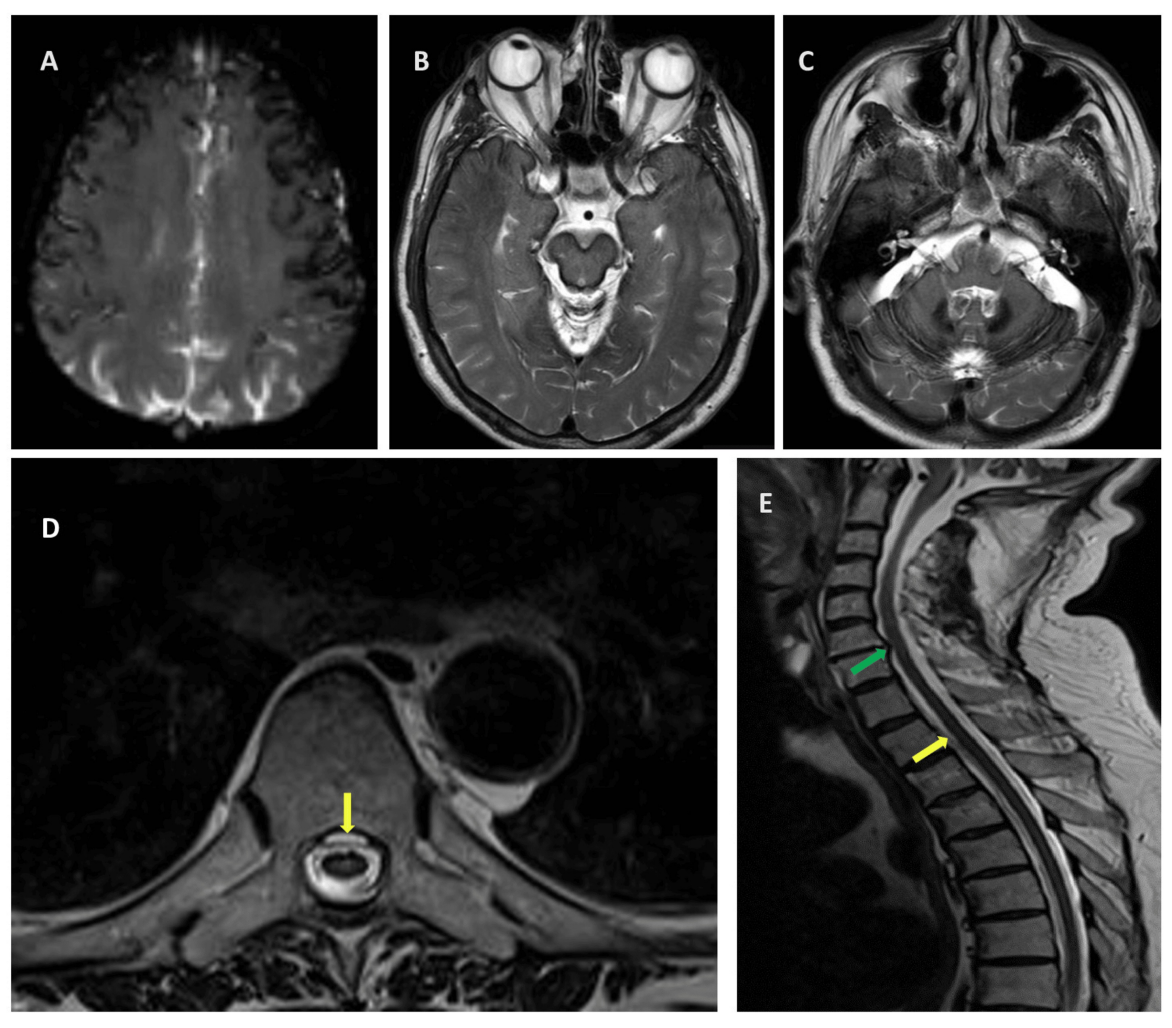
The axial and sagittal brain and spinal MRI from patient Axial gradient echo (A) and T2-weighted (B-C) brain MRI showing signal hypointensity due to hemosiderin deposition along the cerebral convexities (A), around the midbrain (B), and along the cerebellar folia (C). The T2-weighted axial (D) and sagittal (E) images of the cervical and thoracic spine showed ventral epidural fluid collection extending from C6 to T10 (yellow arrows) and disc-osteophyte complex at the C6-7 disc level (green arrow).

On brain magnetic resonance imaging (MRI), axial T2-weighted images and gradient echo sequences showed linear superficial hypointensity in the cerebrum, cerebellum, and brain stem (Figures 1A-1C). The T2-weighted sagittal and axial image of the cervical and thoracic spine (Figures 1D, 1E) showed ventral epidural fluid collection extending from C6 to T10 and disc-osteophyte complex at the C6-7 disc level. Due to the disk-osteophyte complex being clearly identified and both lumbar puncture and CT myelography being invasive procedures, these examinations were not conducted. The patient's complete blood count, prothrombin time, activated partial thromboplastin time, serum ferritin, and iron levels were within normal limits.

C6-C7 microdiscectomy and duraplasty were performed with the anterior approach for the C6-C7 disc-osteophyte complex. At the six-month follow-up after surgery, the patient's neurological examination demonstrated improvement in appendicular ataxia, truncal ataxia, and dysarthria. Although the patient had a wide-based gait, he was able to walk without the need for assistance. Despite improvement in ataxia, hearing loss persisted, and the patient underwent cochlear implant surgery. Hence, postoperative MRIs were not assessable due to artifacts resulting from the cochlear implant surgery. In the second year following the surgery, the patient's ataxia and dysarthria did not show any progression. However, treatment was initiated due to the development of depressive symptoms and aggression.

## Discussion

Superficial siderosis is a progressive chronic disease caused by the deposition of hemosiderin in the subpial space as a result of chronic leakage into the subarachnoid space. Inflammation caused by toxic free iron accumulation in the pia is thought to play an important role in the pathogenesis [1]. The classic clinical triad of superficial siderosis is sensorineural hearing loss, cerebellar ataxia, and myelopathy. Patients may experience headaches indicative of intracranial hypotension. The most common cause of SS is spinal dural defects, as in the presented case. Kumar et al. reported spinal dural defects in 18 of 30 cases with SS that could potentially explain chronic subarachnoid bleeding [3]. A recent study from the United Kingdom noted a spinal dural abnormality in 40 of 65 cases of SS [5]. Similar to our case, most of these patients had a ventrally located longitudinal spinal cerebrospinal fluid (CSF) collection. Ventral epidural fluid collection is typically caused by a disc herniation, often characterized by calcification or an osteophyte that may have a spiculated nature, leading to a dural tear [2,8]. Tumors, radiation, and vascular malformations have also been reported as causes of SS [2,3,9,10].

Blood-sensitive sequences in MRI are the most commonly used imaging methods in the diagnosis of SS. On T2 images, hypointensity is seen in the cerebellar and brain stem, spinal surfaces, cortical sulci, and Sylvian or interhemispheric fissures. Cerebellar and spinal atrophy are also common. In the presented case, the etiology and site of dural defects were identified by spinal CT showing ventral epidural fluid collection extending from C6 to T10, and disc-osteophyte complex seen at the C6-7 disc level on spinal MRI. Since there was no lesion in another region that could have produced CSF leakage, it was suggested that this level was the source of CSF leakage, and the patient was referred to surgery without further investigation with CT myelography. The benefits of surgical intervention for repairing the dural defect in SS patients have been reported in the literature [6,7,11]. Although the importance of early diagnosis and surgical treatment in SS is emphasized in the literature, the presented case proved that patients with a long-term disease history can also benefit from surgical treatment. Chelation therapy with deferiprone is not preferred in routine treatment because the benefit of chelation therapy has not been demonstrated in the existing small number of case series; furthermore, serious side effects such as neutropenic sepsis have been reported with chelation [12].

## Conclusions

Superficial siderosis is a slowly progressive disease, but clinical progression can be prevented, and clinical improvement can be achieved with the detection of the source of bleeding and appropriate surgical interventions even after a long disease course. In patients with SS who have not been treated for a long time, the clinical benefit may be limited due to irreversible neuronal damage. Despite observational data suggesting that identifying and treating causative structural defects may be beneficial, large-scale controlled trials are lacking.
